# Execution of Diagnostic Testing Has a Stronger Effect on Emergency Department Crowding than Other Common Factors: A Cross-Sectional Study

**DOI:** 10.1371/journal.pone.0108447

**Published:** 2014-10-13

**Authors:** Takahisa Kawano, Kei Nishiyama, Hiroyuki Hayashi

**Affiliations:** 1 Department of Emergency Medicine, University of Fukui Hospital, Fukui, Japan; 2 Department of Primary Care and Emergency Medicine, Kyoto University Graduate School of Medicine, Kyoto city, Japan; 3 Department of General Medicine, University of Fukui Hospital, Fukui, Japan; University of Utah School of Medicine, United States of America

## Abstract

**Study Objective:**

We compared the effects of execution of diagnostic tests in the emergency department (ED) and other common factors on the length of ED stay to identify those with the greatest impacts on ED crowding.

**Methods:**

Between February 2010 and January 2012, we conducted a cross-sectional, single-center study in the ED of a large, urban, teaching hospital in Japan. Patients who visited the ED during the study period were enrolled. We excluded (1) patients scheduled for admission or pharmaceutical prescription, and (2) neonates requiring intensive care transferred from other hospitals. Multivariate linear regression was performed on log-transformed length of ED stay in admitted and discharged patients to compare influence of diagnostic tests and other common predictors. To quantify the range of change in length of ED stay given a unit change of the predictor, a generalized linear model was used for each group.

**Results:**

During the study period, 55,285 patients were enrolled. In discharged patients, laboratory blood tests had the highest standardized β coefficient (0.44) among common predictors, and increased length of ED stay by 72.5 minutes (95% CI, 72.8–76.1 minutes). In admitted patients, computed tomography (CT) had the highest standardized β coefficient (0.17), and increased length of ED stay by 32.7 minutes (95% CI, 40.0–49.9 minutes). Although other common input and output factors were significant contributors, they had smaller standardized β coefficients in both groups.

**Conclusions:**

Execution of laboratory blood tests and CT had a stronger influence on length of ED stay than other common input and output factors.

## Introduction

### Background

Emergency department (ED) crowding represents a serious problem in emergency medicine. In Japan, 13,000 patients needing critical care experienced ambulance diversion at least four times during one emergency transport; nearly one-quarter of this ambulance diversion was caused by ED crowding. [1] Further, ED crowding does not only cause this ambulance diversion, but also delays treatment in the ED, which adversely affects patient outcomes. Indeed, increased mortality has been observed in patients admitted from the ED when ED crowding is severe. [2].

Recently, diagnostic tests in ED were recognized as one of major contributors to ED crowding. [3] For example, when laboratory tests are ordered for ED patients, an additional 80 minutes are reportedly added to the length of ED stay. [4] However, other contributors to ED crowding have been identified. For example, entry overload contributed to ED crowding in Australia. [5] High hospital bed occupancy can block admissions from the ED and prolong patient stay in the ED. [6] Additionally, patient age is an independent contributor to ED crowding, with older patients staying longer than young patients. [7] The glut of factors contributing to ED crowding has precluded an effective prioritization of interventions to reduce the problem. To establish effective interventions, identifying contributing factors and understanding the extent of their contributions to ED crowding is necessary.

We hypothesized that execution of diagnostic tests in ED has a close relation to ED crowding both in admitted and discharged patients. Here, we assessed the influence of diagnostic tests and other common contributors on the length of ED stay in each group toward the identification of factors whose negative impact on ED stay can be mitigated through appropriate policies and procedures.

## Methods

### Study design and setting

We conducted a cross-sectional, single-center study in the ED of the Fukui Prefectural Hospital in Fukui, Japan. The Fukui Prefectural Hospital, a large teaching hospital in an urban setting, has the largest ED in the mid-Fukui Prefecture area, where approximately 380,000 residents live. Another large academic hospital with an ED is located in this area. These hospitals serve both urban and rural communities in an area of 830 km^2^ with a population density of 450 persons per km^2^. The area has 7 fire departments and 7 emergency dispatch centers. Fukui Prefectural Hospital has 1,082 beds and 14 intensive care unit beds. The main treatment location at the ED has four beds for serious cases or patients being resuscitated. There are also four examination rooms for low-acuity patients and four beds for observation. Almost all patients who visited this ED were self-referred. The ED accepts all patients regardless of their age, including pediatric patients. The ED performs simple radiologic studies, laboratory blood tests, urinalysis, CT, MRI, and electrocardiography. Laboratory blood tests are analyzed in a central laboratory; a point-of-care test for blood gas analysis, cardiac troponin level, and β-human chorionic gonadotropin is available, and for admitted patients, laboratory blood tests were conducted routinely. General staffing comprises six residents, one attending physician, and three nurses per shift. Residents are responsible for the first encounter with almost all patients who come to the ED. However, the attending physician begins evaluation of high-acuity patients without awaiting evaluation by residents. All other specialist consultants are available when necessary.

### Selection of participants

Patients who visited the ED at Fukui Prefectural Hospital from February 1, 2010 to January 31, 2012 were enrolled in our study. We excluded (1) patients who were scheduled for admission or pharmaceutical prescription, and (2) neonates requiring intensive care who were transferred from other hospitals. The regular reception desks at our hospital are closed on weekends. Patients arriving for scheduled admission or pharmaceutical prescription visit the reception desk at the ED. Patients needing pharmaceutical prescription mainly receive daily intravenous antibiotic administration at outpatient clinics. These patients quickly depart the ED without any evaluation or treatment, so the length of ED stay of these patients is small. Neonates requiring intensive care who are transferred from another hospital typically stay in the ED during registration only, so their length of ED stay is also small. Because these patients rarely consume any ED resources beyond registration, we excluded them. We classified participants into admitted and discharged patient groups to consider patient acuity in this study.

### Outcomes

Study outcome was the length of ED stay, one of the most common proxy measures of overcrowding. [8] The length of ED stay was defined as the time, in minutes, from registration in the ED to ED departure.

### Definitions

We chose 14 variables for assessment in this study following review of the literature. To understand the causes of ED crowding, the input-throughput-output conceptual model has been accepted. [9] According to the model, the 14 variables in this study were classified into four categories; input factors, or those relating to patient entry to the ED; throughput factors, or those relating to ED care processes; output factors, or those relating to the disposition of patients from the ED by discharge or hospital admission; and other factors. Input factors in this study included mode of arrival, number of walk-in, and the number of ambulance arrivals. Throughput factors in this study included diagnostic tests (laboratory blood tests, radiography, CT, MRI, ultrasonography, and electrocardiography). Output factors in this study included hospital bed occupancy and ED boarding. Patient age, arrival time and percentage of hospital admissions were included as other factors.

Mode of arrival represented the method of patient entry to the ED, by ambulance or walk-in. [10] Number of walk-ins was defined as the number of walk-in patients arriving in the ED each hour. The number of ambulance arrivals at the ED was calculated hourly. Laboratory blood tests were defined as those blood tests analyzed in the central laboratory; point-of-care blood tests were excluded. Hospital bed occupancy was defined as the percentage of hospital beds occupied daily. [6] ED boarding was defined as the number of patients at each hour who stayed in the ED for more than 1 hour after admission by an ED physician. [6] Arrival time was classified into 3 shifts (08∶00–15∶59, 16∶00–23∶59, and 00∶00–07∶59) to assess the effect of staffing on the length of ED stay. The percentage of admissions represented the percentage of hospital admissions originating from the ED per day. [11].

### Measurement

We obtained data from the electronic medical record (EMR) system and the electronic hospital administrative data system of our hospital. Patient age, arrival time, time of decision to admit, departure time, disposition of patient, mode of arrival, and execution of any diagnostic tests were obtained from the EMR of each patient. This record is considered to be accurate because every patient account is generated from it. When a patient needs to be admitted, the physician orders admission through EMR. Nurses follow the necessary procedures after this order and transfer patients to hospital wards. If patients have to wait in the ED for the arrangement of a hospital bed, nurses record these patients and provide care to them appropriately. We obtained this nursing record to calculate ED boarding. ED clerks obtained the number of available hospital beds at 09∶00 each day from the electronic hospital administrative data system and calculated hospital bed occupancy. We could not obtain hourly hospital bed occupancy information.

### Statistical analysis

To compare influence of diagnostic tests and other predictors on length of ED stay of admitted and discharged patients, multivariate linear regression was performed in each group (see database in Appendix S1). Length of ED stay in this study was not normally distributed, which corresponded to findings of previous studies (Absolute skew value 2.24, absolute kurtosis 9.54). [6] Therefore, we applied the natural log transform to length of ED stay. For sample sizes greater than 300, the distribution is considered normal if the absolute skew value was less than 2 and the absolute kurtosis less than 7. [12] Thus, the log-transformed length of ED stay in this study was considered as normally distributed (absolute skew value 0.42, absolute kurtosis 1.02). We subsequently obtained the standardized partial regression coefficient (standardized β coefficient) of each predictor to compare their influences on length of ED stay. Each predictor was measured in different units of measurement and had a different variance, so we could not compare each regression coefficient in original units in this model. Therefore, to make their variance equal, the model was applied to standardized predictors, and we accessed the contribution of predictors to LOS using their standardized β coefficients. We tested this model for multicolinearity by measuring the variance inflation factor. The inflation factor of laboratory blood tests in admitted patients was more than 10 and considered to have multicolinearity. Thus, laboratory blood tests were excluded from the analysis of admitted patients.

To quantify the range of change in length of ED stay of each group given a unit change in the value of the predictor, a generalized linear model (GLM) with a log-link function and gamma distribution was conducted. GLM is commonly used to analyze length of ED stay because of its skewedness. [3], [6] Laboratory blood tests in admitted patients also demonstrated multicolinearity in this model by measurement of the variance inflation factor, and were excluded from the analysis of admitted patients.

Descriptive analyses were conducted with the use of frequency tabulations. We described the medians and interquartile ranges (IQR) for all variables. All analyses were conducted with STATA version 12.1 (STATA Corp LP, College Station, TX). The authors had full access to the data and take responsibility for its integrity. All authors have read and agreed to the content of the manuscript.

### Ethical Statement

The study protocol accorded with the guidelines for epidemiologic studies issued by the Ministry of Health, Labor and Welfare of Japan. [13] The ethics committees in Fukui Prefectural Hospital approved the research protocol. According to the guidelines, the requirement of written informed consent was allowed to be waived by the ethics committees, because our study didn’t include any personally identifiable information. [13], [14] We posted the poster about our study protocol on the bulletin board in the ED instead of written informed consent.

## Results

### Patient characteristics

**Figure 1:**
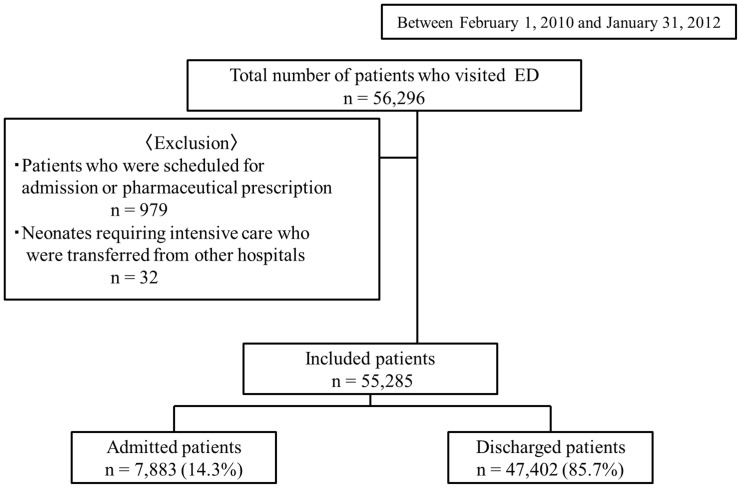
Patient flow diagram. ED, emergency department.

During the study period, 56,296 patients visited the ED. Of these, 979 patients who were scheduled to visit the ED for admission or pharmaceutical prescription, as well as 32 newborn babies who were transferred from other hospitals, were excluded. The study therefore included 55,285 (98.2%) of the total patients who visited the ED. Of the included patients, 7,883 (14.3%) were admitted to the hospital, and 47,402 (85.7%) were discharged (Figure 1).

The median (quartiles) age of included patients was 35 years [interquartile range (IQR), 14–62 years]. Of the included patients, 14.0% were transported to the ED by ambulance (Table 1). The median length of ED stay for all patients was 91 minutes (IQR, 54–154 minutes). The median lengths of ED stay for the discharged and admitted patients were 83 minutes (IQR, 51–137 minutes) and 162 minutes (IQR, 113–234 minutes), respectively.

**Table 1:** Patient demographics and primary outcome.

	Total
	(n = 55,285)
Median patients age, y (IQR)	35 (14−62)
Walk-in patients, n	48,558
per day, n (IQR)	55 (46−80)
08∶00 to 15∶59, n (IQR)	15 (12−32)
16∶00 to 23∶59, n (IQR)	32 (27−39)
0∶00 to 07∶59, n (IQR)	9 (6−12)
Ambulance, n	6760
per day, n (IQR)	9 (7−1)
08∶00 to 15∶59, n (IQR)	4 (2−5)
16∶00 to 23∶59, n (IQR)	3 (2−5)
0∶00 to 07∶59, n (IQR)	1 (1−3)
Admissions, n	7,843
per day, n (IQR)	10 (8−13)
Percentage of admission, % (IQR)	15.4 (11.5−19.1)
Hospital bed occupancy	
per day, % (IQR)	74.1 (70.6−75.8)
ED boarding	
per day, n (IQR)	2 (1−3)
Deaths, n (%)	185 (1.6)
Median Length of ED stay	
Total patients, min (IQR)	92 (55−154)
Discharged patients, min (IQR)	83 (51−137)
Admitted patients, min (IQR)	162 (113−234)

IQR, interquartile range; ED, emergency department.

Laboratory blood tests were the most frequent examination ordered in the ED; 20,125 patients (36.4%) received these tests, and all admitted patients had laboratory blood tests in the ED (Table 2). Of other diagnostic tests available, radiographies, CTs, ultrasonographies, electrocardiograms, and MRIs were conducted on 16,820 (30.4%), 11,696 (21.2%), 10,794 (19.5%), 10,663 (19.3%), and 1,360 patients (2.5%), respectively (Table 2). Admitted patients underwent these diagnostic tests in ED more frequently than discharged patients.

**Table 2:** The number of diagnostic tests in ED.

	Total patients	Admitted patients	Discharged patients	
Number of diagnostic examination in ED	(n = 55,285)	(n = 7,843)	(n = 47442)	P value
X-ray, n	16,820	6778	9905	
proportion of examined patients per day, % (IQR)	31 (26−36)	88 (80−100)	20.9 (17.3−25.5)	<0.01
Computed tomography, n	11,696	4357	7326	
proportion of examined patients per day, % (IQR)	22 (17−26)	55.6 (44.4−6.7)	16.1 (11.9−20.6)	<0.01
Magnetic resonance imaging, n	1,360	653	706	
proportion of examined patients per day, % (IQR)	2 (1−4)	7.7 (0−13.3)	1.4 (0−2.4)	<0.01
Blood examination, n	20,125	7843	12016	
proportion of examined patients per day, % (IQR)	37 (31−43)	100 (100−100)	26.4 (21.1−31.4)	–
Ultrasonography, n	10,794	2693	7998	
proportion of examined patients per day, % (IQR)	20 (15−25)	33.3 (25−4.4)	17.2 (13.6−2.0)	<0.01
Electrocardiogram, n	10,663	5523	5143	
proportion of examined patients per day, % (IQR)	20 (15−25)	71.4 (60.8−81.3)	11.2 (7.8−15.1)	<0.01

IQR, interquartile range.

### Discharged patients

We compared the influence of input, throughput, and output factors on length of stay in the ED through multivariate regression in discharged patients (Table 3). The cross correlation of included variables in this analysis was described in table 4. In discharged patients, laboratory blood testing, a throughput factor, had the highest standardized β coefficient (0.44) among possible predictors and increased length of ED stay by 74.5 minutes. CT and radiography-throughput factors-also had high standardized β coefficients (0.17 and 0.16) and increased length of ED stay by 32.7 and 27.0 minutes respectively. Number of walk-ins (common input factor) and hospital bed occupancy (common output factor) were statistically significant contributors in log-transformed regression analysis. However, these factors had smaller absolute standardized β coefficients than the diagnostic tests (0.09 and 0.01). Number of walk-ins increased length of ED stay by only 1.8 minutes per increase of the number of hourly walk-in patients.

**Table 3:** Comparison of the effects of input, throughput, and output factors on the length of ED stay for discharged patients.

	Discharged patients (n = 47442)			
Characteristic	standardized β coefficient	95% CI	Additional length of stay, minutes	95% CI
Input factors				
Mode of arrival				
Walk-in	Reference			
Ambulance	−0.02*	−0.03–−0.01	2.4	<−0.5−4.9
Number of walk-in patients	0.09*	0.08−0.97	1.8*	1.6−2.0
Number of ambulance arrivals	0.02*	0.02−0.03	2.6*	1.7−3.4
Throughput factors				
No diagnostic test	Reference			
Radiography	0.17*	0.16−0.17	27.0*	25.6−28.6
Computed tomography	0.16*	0.16−0.17	32.7*	31.0−34.5
Magnetic resonance imaging	0.06*	0.05−0.07	38.3*	33.4−43.2
Ultrasonography	0.08*	0.08−0.09	13.6*	12.0−15.2
Laboratory blood tests	0.44*	0.44−0.45	74.5*	72.8−76.1
Electrocardiogram	<0.01	<−0.01−0.01	1.6	−0.5−3.7
Output factors				
Bed occupancy	−0.01*	−0.02–−0.01	−0.1	−0.2−0.7
ED boarding	0.01	<−0.01−0.01	0.7	−0.5−2.0
Other factors				
Patient age	0.04*	0.03−0.05	0.2*	0.2−0.2
Arrival				
08∶00–15∶59	Reference			
16∶00–23∶59	<−0.01	−0.01–<0.01	−3.0*	−4.4–−1.8
0∶00–07∶59	<0.01	<−0.01−0.01	− 0.1	−1.9−1.9
Percentage of admissions	<0.01	<−0.01–<0.01	0.2*	0.1−0.3

*,P<0.05; 95% CI, 95% confidence intervals; ED, emergency department.

**Table 4:** Cross correlation of input, throughput, and output factors on the length of ED stay for discharged patients.

Characteristic	Ambulancearrival	Number ofwalk-inpatients	Number ofambulancearrivals	Radiography	Computedtomography	Magneticresonanceimaging	Ultrasonography	Laboratorybloodtests
Ambulance arrival	1							
Number of walk-in patients	−0.0901	1						
Number of ambulancearrivals	0.3521	0.195	1					
Radiography	0.1971	−0.0441	0.0757	1				
Computed tomography	0.2536	−0.0885	0.0764	0.1627	1			
Magnetic resonanceimaging	0.0833	−0.048	0.0295	0.0444	0.1961	1		
Ultrasonography	0.1654	−0.114	0.0283	0.0989	0.1905	−0.0144	1	
Laboratory blood tests	0.2803	−0.0999	0.0893	0.2218	0.3084	0.1246	0.267	1
Electrocardiogram	0.2605	−0.0996	0.0741	0.2649	0.2411	0.1291	0.1978	0.4087
Bed occupancy	0.0227	−0.2207	−0.0302	0.01	0.0462	0.0276	0.0413	0.0358
ED boarding	−0.0127	0.0506	0.0056	−0.0087	−0.0083	−0.0103	−0.0157	−0.0156
Patient age	0.1659	−0.0644	0.0476	0.1883	0.2812	0.1332	0.1321	0.2912
Arrival 16∶00–23∶59	−0.0644	0.1643	0.0188	−0.034	−0.0665	−0.0466	−0.0478	−0.059
Arrival 0∶00–07∶59	0.0479	−0.3665	−0.0926	−0.0026	0.0455	0.0126	0.1082	0.0499
Percentage ofadmissions	0.0542	−0.2895	0.0557	0.031	0.0568	0.029	0.0499	0.057
	Electrocardiogram	Bed occupancy	ED boarding	Patient age	Arrival 16∶00–23∶59	Arrival 0∶00–07∶59	Percentage of admissions	
Electrocardiogram	1							
Bed occupancy	0.0399	1						
ED boarding	−0.0091	−0.0069	1					
Patient age	0.3324	−0.0127	−0.0049	1				
Arrival 16∶00–23∶59	−0.0646	0.1164	0.0122	−0.1337	1			
Arrival 0∶00–07∶59	0.0482	0.0063	−0.095	0.0385	−0.4229	1		
Percentage ofadmissions	0.0569	0.2707	0.0683	0.0352	0.0986	0.024	1	

ED, emergency department.

### Admitted patients

In admitted patients, laboratory blood tests were omitted due to multicolinearity in both log-transformed regression analysis and GLM. The cross correlation of included variables for the admitted patient analysis was also described in table 6. CT had the highest standardized β coefficient (0.25) among possible predictors and increased length of ED stay by 44.9 minutes (Table 5). Number of walk-ins and ED boarding (common output factors) were also statistically significant contributors in log-transformed regression analysis. However, these factors had smaller standardized β coefficients than CT (0.07 and 0.02). Number of walk-ins also increased length of ED stay by only 2.3 minutes per increase in the number of hourly walk-in patients.

**Table 5:** Comparison of the effects of input, throughput, and output factors on the length of ED stay for admitted patients.

	Admitted patients (n = 7,843)			
Characteristic	standardized β coefficient	95% CI	Additional length of stay, minutes	95% CI
Input factors				
Mode of arrival				
Walk-in	Reference			
Ambulance	−0.20*	−0.22–−0.17	−38.6*	−44.1–−3.1
Number of walk-in patients	0.06*	0.04−0.09	2.3*	1.4−3.1
Number of ambulance arrivals	0.01	−0.01−0.04	1.2	−2.0−4.3
Throughput factors				
No diagnostic test	Reference			
Radiography	0.06*	0.03−0.08	2.5	−4.7−9.7
Computed tomography	0.25*	0.23−0.27	44.9*	40.0−49.9
Magnetic resonance imaging	0.14*	0.12−0.16	53.9*	45.6−62.3
Ultrasonography	0.07*	0.04−0.09	12.5*	7.6−17.4
Laboratory blood tests	omitted			
Electrocardiogram	0.01	<−0.01−0.04	−0.4	−0.7−0.5
Output factors				
Bed occupancy	−0.01	−0.03−0.01	−0.1	−0.7−0.5
ED boarding	0.02*	<0.01−0.04	2.2	−2.5−6.8
Other factors				
Patient age	0.01	<−0.02−0.03	0.1*	<0.1−0.2
Arrival				
08∶00–15∶59	Reference			
16∶00–23∶59	−0.05*	−0.08–−0.03	−12.9*	−17.8–−8.0
0∶00–07∶59	0.08*	0.06−0.11	36.5*	29.2−43.7
Percentage of admissions	0.02	<−0.01−0.04	0.3	−0.1−0.8

*, P<0.05; 95% CI, 95% confidence intervals; ED, emergency department.

**Table 6:** Cross correlation of input, throughput, and output factors on the length of ED stay for admitted patients.

Characteristic	Ambulancearrival	Number ofwalk-inpatients	Number ofambulancearrivals	Radiography	Computedtomography	Magneticresonanceimaging	Ultrasonography	Electrocardiogram
Ambulance arrival	1							
Number of walk-inpatients	−0.1181	1						
Number of ambulancearrivals	0.501	0.1558	1					
Radiography	0.1261	−0.0527	0.0753	1				
Computed tomography	0.1214	−0.0469	0.0505	0.2432	1			
Magnetic resonanceimaging	0.0679	−0.0302	0.0221	0.0534	0.1934	1		
Ultrasonography	0.1107	−0.0884	0.0352	0.1471	0.2145	−0.0848	1	
Electrocardiogram	0.1731	−0.0906	0.0821	0.4485	0.2731	0.1073	0.2004	1
Bed occupancy	0.0496	−0.2389	−0.0038	0.0211	0.0146	0.0153	0.0725	0.0162
ED boarding	−0.0326	0.0525	−0.01	−0.0146	−0.0135	−0.0007	−0.028	−0.0271
Patient age	0.2223	−0.0771	0.1286	0.3038	0.2484	0.1231	0.1417	0.4673
Arrival 16∶00–23∶59	−0.0437	0.2523	−0.0013	−0.1196	−0.0502	−0.0541	−0.0341	−0.1117
Arrival 0∶00–07∶59	0.076	−0.3022	−0.0553	0.0336	0.0607	0.0393	0.1084	0.0719
Percentage ofadmissions	0.071	−0.2799	0.108	0.0001	0.0172	0.016	0.0244	0.0183
	Bed occupancy	ED boarding	Patient age	Arrival 16∶00–23∶59	Arrival 0∶00–07∶59	Percentage of admissions		
Bed occupancy	1							
ED boarding	0.0019	1						
Patient age	0.0295	−0.0161	1					
Arrival 16∶00–23∶59	0.0364	0.0197	−0.119	1				
Arrival 0∶00–07∶59	−0.0058	−0.0889	0.011	−0.3228	1			
Percentage ofadmissions	0.2564	0.0562	0.0611	0.0268	−0.0246	1		

ED, emergency department.

### Limitations

Several limitations existed in this study. First, we did not classify participants in this study according to severity and complexity of their medical condition, because we could not obtain triage results. The severity and complexity likely affected physicians’ decisions regarding diagnostic testing in the ED. This is the most important limitation in this study. Second, the findings were obtained from a single institution over two years, which might reduce the generalizability of the conclusions. However, our approach corresponds to that of previous studies of ED crowding in a single site; [8] therefore, our findings may be generalizable, especially to other teaching hospitals. Third, our institution is a teaching facility, requiring emergency physicians to evaluate and treat patients after initial evaluation by resident physicians. This additional step might extend the ED stay. [15] Fourth, although ambulance diversion and patients leaving before evaluation are important measures in ED crowding, we did not include them in this study because we did not measure them. Fifth, staffing in ED and the number of consultations were also not measured. [11] We cannot exclude possible bias from these unmeasured factors. Sixth, we could not obtain hourly hospital bed occupancy due to insufficiency of our electronic hospital administrative data system. Ideally, data analysis would be performed according to the precise hospital bed occupancy at particular times because hospital bed occupancy changes every hour. However, previous studies have also used daily hospital bed occupancy and found an association with length of ED stay. [11], [16] Finally, we could not distinguish necessary diagnostic tests from unnecessary tests in our study. These unnecessary diagnostic tests might contribute to extended patient stay in the ED.

## Discussion

In this analysis of input, output, and throughput factors contributing to length of ED stay, laboratory blood testing had the strongest influence on length of ED stay in discharged patients, increasing it by 74.5 minutes compared to those discharged patients who did not have blood tests. CT testing had a strong influence on length of ED stay in admitted patients, increasing it by 44.9 minutes compared to that of admitted patients who did not undergo CT. Input and output factors also had significant influences on length of ED stay. However, their contributions did not have as great an impact as these diagnostic tests. Our findings will enable ED physicians to prioritize the contribution of input, throughput, and output factors to ED crowding and identify diagnostic tests that have a close relationship with length of ED stay both in discharged and admitted patients.

While many input, throughput, and output factors contributed to ED crowding, hospital bed insufficiency has been considered a major contributor to ED crowding. [17] However, the strength of contribution of this hospital bed insufficiency to ED crowding was not compared with those of other factors in any study. Indeed, ED physicians could not effectively set priorities of countermeasures against ED crowding because a lot of recommendations about solutions for input, throughput, and output factors were treated equally. [17] In our study, comparing the effect of input, throughput, and output factors on length of ED stay revealed that diagnostic tests, especially laboratory blood tests and CT, have a stronger effect on ED stay than other factors. Our findings suggest that interventions aimed at improving efficacy of diagnostic tests, especially laboratory blood tests and CT, should be explored.

The finding of the association between diagnostic testing and ED stay may result from two phenomena: the complex process of these diagnostic tests, and the use of unnecessary testing. First, the process of acquiring diagnostic tests takes many steps. For example, laboratory blood tests require placing the order, sampling patient blood, transporting it to the laboratory, analyzing it, and reporting of the results to the physician. This process, namely turnaround time of diagnostic tests, is considered as an important determinant of length of ED stay. [18] Thus, turnaround time of diagnostic tests might contribute to extended stay in ED of patients who had these tests in our study. Second, in general, physicians select diagnostic tests for patients who were considered to be high acuity or of complex medical condition. [19], [20] However, these tests do not always yield additional value to management of these patients. [21] For example, one study reported that physicians established and used guidelines to select diagnostic tests for patients with an acute limp. This practice reduced the number of unnecessary tests, and the length of ED stay of these patients decreased. [22] Our study implies the improvement of turnaround time of laboratory blood tests and CT and the reduction of unnecessary testing might be effective solutions for ED crowding.

Numerous countermeasures on ED crowding have been investigated. Redirection of low-acuity patients to alternative facilities and ambulance diversion were studied as countermeasures controlling ED volume. [23], [24] However, our findings suggest that restrictions on input factors may not be most effective to reduce the length of ED stay. An increase of one in the number of walk-in patients and ambulance arrivals prolonged patient stay in ED by only 1.8 and 2.6 minutes respectively; however, the execution of laboratory blood tests added another 74 minutes to stay of discharged patients. Another proposed countermeasure against ED crowding is the use of observation units to improve admission flow from the ED. [25] Once again, however, output factors may not have as strong an influence on length of ED stay as throughput factors. Our findings suggest that countermeasures on input and output factors might not be as effective as countermeasures on diagnostic tests.

Our study has some weaknesses. First, input factors in this study may have a smaller effect on the length of ED stay than they would in large EDs. However, large EDs with annual patients volumes of over 60,000 account for only 11% of total EDs in U.S. [26] Annual patient volume of our ED matches that of mid-sized EDs in the U.S., where annual patient volumes are 20,000–40,000; these EDs comprise 25% of total EDs in the U.S. [26] Indeed, the effect of input factors on the length of ED stay increases with increased patient volume, but this trend is observed at both large and small EDs. [26] This, the findings of our study may be most generalizable to mid- and small-sized EDs. Second, the hospital bed occupancy in this study was not as high as that of previous studies. [6], [27] However, an effect of bed occupancy on the length of ED stay was observed in EDs where median hospital bed occupancy was around 70%. [11], [16] The effect of hospital bed occupancy seems to be limited in this study and our findings should be validated in the EDs with higher hospital bed occupancy.

## Conclusion

In our study, performance of diagnostic tests had a stronger relationship to increased length of ED stay than other common input and output factors both in discharged and admitted patients. In addition to other known factors that can contribute to ED crowding, emergency providers should develop an improved sensitivity to the time costs associated with the diagnostic testing they order and how these can detrimentally affect patient length of stay.

## Supporting Information

Appendix S1 database.
(XLSX)
